# Supplementary material for: Resolving the full spectrum of human genome variation using Linked-Reads
Source: Genome Res. 2019 Apr;29(4):635–45. doi: 10.1101/gr.234443.118 (PMC6442396; doi:10.1101/gr.234443.118)
Supplement: Supplemental Material [file supp_gr.234443.118_Supplemental_longranger-2.2.zip › longranger-2.2.tar.bz2!/lr2.2-public/lib/stan/cmdstan/stan/doc/api/html/math_2constants_8hpp.html]

Stan: src/stan/math/constants.hpp File Reference


|  |  |
| --- | --- |
|  | Stan  2.5.0  probability, sampling & optimization |


- Main Page
- Related Pages
- Namespaces
- Classes
- Files

- File List
- File Members

All Classes Namespaces Files Functions Variables Typedefs Enumerations Enumerator Friends Macros Pages

- src
- stan
- math

Namespaces |
Functions |
Variables

constants.hpp File Reference

`#include <boost/math/constants/constants.hpp>`

Go to the source code of this file.

|  |  |
| --- | --- |
| Namespaces | |
|  | stan |
|  | Probability, optimization and sampling library. |
|  | |
|  | stan::math |
|  | Matrices and templated mathematical functions. |
|  | |

|  |  |
| --- | --- |
| Functions | |
| double | stan::math::pi () |
|  | Return the value of pi. More... |
|  | |
| double | stan::math::e () |
|  | Return the base of the natural logarithm. More... |
|  | |
| double | stan::math::sqrt2 () |
|  | Return the square root of two. More... |
|  | |
| double | stan::math::log10 () |
|  | Return natural logarithm of ten. More... |
|  | |
| double | stan::math::positive\_infinity () |
|  | Return positive infinity. More... |
|  | |
| double | stan::math::negative\_infinity () |
|  | Return negative infinity. More... |
|  | |
| double | stan::math::not\_a\_number () |
|  | Return (quiet) not-a-number. More... |
|  | |
| double | stan::math::machine\_precision () |
|  | Returns the difference between 1.0 and the next value representable. More... |
|  | |

|  |  |
| --- | --- |
| Variables | |
| const double | stan::math::E = boost::math::constants::e<double>() |
|  | The base of the natural logarithm, . More... |
|  | |
| const double | stan::math::SQRT\_2 = std::sqrt(2.0) |
|  | The value of the square root of 2, . More... |
|  | |
| const double | stan::math::INV\_SQRT\_2 = 1.0 / SQRT\_2 |
|  | The value of 1 over the square root of 2, . More... |
|  | |
| const double | stan::math::LOG\_2 = std::log(2.0) |
|  | The natural logarithm of 2, . More... |
|  | |
| const double | stan::math::LOG\_10 = std::log(10.0) |
|  | The natural logarithm of 10, . More... |
|  | |
| const double | stan::math::INFTY = std::numeric\_limits<double>::infinity() |
|  | Positive infinity. More... |
|  | |
| const double | stan::math::NEGATIVE\_INFTY = - std::numeric\_limits<double>::infinity() |
|  | Negative infinity. More... |
|  | |
| const double | stan::math::NOT\_A\_NUMBER = std::numeric\_limits<double>::quiet\_NaN() |
|  | (Quiet) not-a-number value. More... |
|  | |
| const double | stan::math::EPSILON = std::numeric\_limits<double>::epsilon() |
|  | Smallest positive value. More... |
|  | |
| const double | stan::math::NEGATIVE\_EPSILON = - std::numeric\_limits<double>::epsilon() |
|  | Largest negative value (i.e., smallest absolute value). More... |
|  | |
| const double | stan::math::LOG\_PI\_OVER\_FOUR = std::log(boost::math::constants::pi<double>()) / 4.0 |
|  | |
| const double | stan::math::TWO\_OVER\_SQRT\_PI = 2.0 / std::sqrt(boost::math::constants::pi<double>()) |
|  | |
| const double | stan::math::NEG\_TWO\_OVER\_SQRT\_PI = -TWO\_OVER\_SQRT\_PI |
|  | |
| const double | stan::math::INV\_SQRT\_TWO\_PI = 1.0 / std::sqrt(2.0 \* boost::math::constants::pi<double>()) |
|  | |


---

[ Stan Home Page ]

*© 2011–2014,
Stan Development Team.*
